# Serosurvey for dengue virus infection among pregnant women in the West Nile virus enzootic community of El Paso Texas

**DOI:** 10.1371/journal.pone.0242889

**Published:** 2020-11-30

**Authors:** Douglas M. Watts, Cynthia M. Rodriguez, Pedro M. Palermo, Veronica Suarez, Susan J. Wong, Jeanette Orbegozo, Alan P. Dupuis, Laura D. Kramer, Fernando J. Gonzalez, Gilbert A. Handel

**Affiliations:** 1 Department of Biological Science, University of Texas at El Paso, El Paso, Texas, United States of America; 2 Diagnostic Immunology Laboratory, Wadsworth Center, New York State Department of Health, Albany, NY, United States of America; 3 Arbovirus Laboratory, Wadsworth Center, New York State Department of Health, Slingerlands, NY, United States of America; 4 Department of Public Health, El Paso, Texas, United States of America; 5 Paul L. Foster School of Medicine, El Paso, Texas, United States of America; Instituut voor Tropische Geneeskunde, BELGIUM

## Abstract

All 4 dengue viruses (DENV) cause sporadic outbreaks of human disease in the Rio Grande Valley along the US-Mexico border. In addition, West Nile virus (WNV) is enzootic in most border communities, and is the only arbovirus known to cause human disease in the El Paso, Texas community. In an effort to determine if DENV were also endemic in the El Paso community, a serosurvey was conducted among mothers at the time of delivery of their babies in selected hospitals. Cord-blood plasma samples obtained from mothers were tested for DENV antibody by an enzyme-linked immuno-sorbent assay (ELISA), plaque reduction neutralization test (PRNT) and a multiplex microsphere immunoassay. All DENV antibody positive plasma samples were also tested for WNV antibody by the same assays to consider the possibility that DENV antibody positive samples reflected WNV cross reactive antibody. The results indicated that 0.74% (11/1,472) of the mothers had a previous DENV infection and that 3.3% (48/1,472) had a previous WNV infection. Of these mothers, 0.20% (3/1,472) had antibody to both DENV and WNV as evidence of infection by both viruses. The results indicated that 0.2% (3/1472) of the mothers were positive for antibody to only WNV envelope, thus suggesting an undetermined flavivirus infection. Although 6 of the 11 DENV antibody positive mothers did not have a history of travel to a DENV endemic country, the findings of this survey provided further evidence of local transmission of WNV and suggested the possibility of focal autochthonous transmission of DENV in the El Paso community.

## Introduction

Currently, dengue virus (DENV) serotypes 1, 2, 3, and 4 of the genus *Flavivirus*, family *Flaviviridae* are the cause of more human illness and death than any other arbovirus disease as well as the cause of a major economic burden in endemic countries [1–5]. The four viruses are globally distributed throughout the tropical and subtropical regions and the geographical range continues to increase with the expanding range of *Aedes aegypti*, the primary vector of these viruses [6]. Sporadic outbreaks of dengue fever (DENF) and dengue hemorrhagic fever (DHF) have been reported since 1980 in the Rio Grande Valley of the United States (US)—Mexico (MX) border region, primarily in the urban communities of Matamoros in the Mexican state of Tamaulipas and in Brownsville, TX and surrounding communities [7–9]. All outbreaks on the US side of the border have been attributed to DENV infected travelers returning from visits during epidemics in the bordering Mexican city of Matamoros [10]. Outbreaks of DF have also been reported from 2007 to 2014 along the US–MX border in Sonora, MX, including 93 cases during 2014 in Yuma, Arizona, however, all infections were apparently acquired on the MX side of the border [11]. More recently, a report indicated that 14.1% of a selected cohort of 77 adults had serological evidence of a past DENV infection and 10.2% (5/61) had evidence of a recent DENV 1 and 2 infection during 2015 in the El Paso adjoining sister community of Ciudad Juarez, MX [12]. Although *Ae*. *aegypti* inhabits the entire US border region, with a sporadic distribution pattern for *Aedes albopictus*, autochthonous transmission of DENV has only been reported from urban communities in the Rio Grande Valley on the US side of the border [13, 14].

West Nile virus (WNV) is a zoonotic virus of the genus *Flavivirus*, family *Flaviviridae* transmitted primarily by *Culex species* mosquitoes to wild avians that serve as the virus amplifying host and to man and equine as dead-end hosts [2, 15]. Since the mid-late1990s, outbreaks of West Nile (WN) fever and encephalitis have occurred globally in tropical and temperate regions and the virus is now endemic in Africa, Asia, Australia, Middle East, Europe, and the Americas. Human cases of WNF and encephalitis were first recognized during 2002 in TX, and annual epidemics documented through 2016 have caused a total of 5,254 cases [16, 17]. The first WN human cases were reported during 2003 in El Paso, Texas, where *Culex quinquefasciatus* and *Culex tarsalis are* the primary vectors [16, 18]. Estimates based on passive surveillance in El Paso indicated that 271 cases of WN occurred between 2003 and 2016, with cases occurring during late June to early November, and peaking in August during each year [17, 19]

Although DENV have not been reported to be associated with human infections and/or disease in the El Paso community, such cases and/or infections may not have been detected because of the reliance on passive surveillance to detect symptomatic cases. As an example, the use of passive surveillance during a 1980 dengue outbreak in Brownsville, TX failed to detect any dengue cases in El Paso, but 63 cases were detected by active surveillance.7 Another possibility for not detecting human cases is because DENV, like most arboviruses more commonly cause asymptomatic infections or infections that cause a mild influenza-like illness [20]. As a result, it may be possible that DENV are causing unrecognized human infections in the El Paso community (Watts D.M., unpublished data). Also, the more recent evidence that DENV was endemic in Ciudad Juarez, Mexico, an adjoining urban community to El Paso raised awareness of the possibility that these viruses are endemic in El Paso community [12]. These observations underscores the critical need for implementing and sustaining active surveillance program for understanding the endemic/enzootic arboviruses, as well as for the early detection of introduced viruses. Therefore, the primary aims of this study was to conduct a serosurvey for DENV antibody based on the testing of cord-blood samples of mothers at the time of delivery of newborns to determine if humans were being infected with DENV in the El Paso community. The cord-blood samples were also tested for WNV and ZIKA virus (ZIKV) antibodies because of the possibility that any samples reactive for DENV antibody in serological tests could reflect cross reactions, especially to WNV, the only other flavivirus known to be associated with human infections in the El Paso community.

## Materials and methods

### Viruses

The DENV used in this study included DENV -1 (strain 16007) that was isolated from a patient during 1964 in Thailand and had received 10 passages in *Ae*. *albopictus (*C6/36) cells, DENV—2 strain 16681 that was isolated in 1964 from a patient in Thailand and received 9 passages in C6/36 cells, and DENV 4 (strain 1036) that was isolated from a patient in 1967 in Indonesia and had received 10 passages in C6/36 cells [21]. DENV-3 (prototype strain H87) was isolated in 1956 from a patient in the Philippines and had received 2 passages in C6/36 cells [22]. The DENV 1, 2 and 4 serotypes were provided by the Dept. of Virology, U.S. Naval Medical Research Unit No. 6, Lima, Peru and the DENV 3 was provided by World Reference Center for Emerging Viruses and Arboviruses, University of Texas Medical Branch, Galveston, TX. The WNV used was a third Vero cell passage of strain NY385-99, originally isolated from the liver of a Snowy Owl (*Nyctea scandiaca*) that died at the Bronx Zoo during the 1999 WNV epizootic in New York City [23]. The WNV was provided by the World Reference Center for Emerging Viruses and Arboviruses, University of Texas Medical Branch, Galveston, TX. All of the viruses were used to prepare working stock viruses in C6/36 cells for use to perform the plaque reduction neutralization test (PRNT) to test plasma samples for WNV and DENV antibody and to infect Vero cells for use to prepare WNV and DENV lysate antigens in African Green Monkey (*Chlorocebus species*) kidney epithelial (Vero) cells to test plasma sample for IgG antibody by an enzyme-linked immunoabsorbent assay *(*ELISA).

### Enrollment of study subjects

The decision to enroll pregnant females in this survey at local hospitals in El Paso, TX was because they were considered appropriate for achieving the aim to determine if DENV were endemic in the El Paso community. The rationale was based on availability of convenient cord-blood samples, and observations in endemic countries in Central America and 6 Asian countries indicated that the incidence of dengue cases dud not differ significantly among males and females [24–27]. The study subjects who volunteered to participate in this survey were enrolled under a protocol (#515533–7) approved January 27, 2014 by the Institutional Review Board (IRB) at the University of TX at El Paso (UTEP), TX. Also, a similar IRB protocol was approved by the participating hospitals that included Sierra Medical Center (SMC), Sierra East Medical Center (SES) and the Providence Memorial Hospital (PMH), El Paso, TX. All participants in this study were 18 years or older, and all provided voluntary written informed consent that was witnessed by the study’s clinical coordinator. The study did not involve reporting of retrospective medical records or archived samples

### Indirect immunoglobulin G (IgG) enzyme-linked immune-adsorbent assay (ELISA)

An aliquot of each human plasma sample was tested initially at a 1:100 dilution for DENV IgG antibody by an indirect ELISA [28]. All DENV IgG antibody positive samples were further tested at a 1:100 dilution for WNV IgG antibodies to DENV and WNV by an indirect ELISA described previously [28, 29]. The samples with antibody titers that had a 4-fold or greater titer between DENV and WNV were considered as presumptive evidence of virus specific type antibody, and those samples with equal titers or less than a 4-fold difference reflected possible infection by both WNV and/or DENV, or cross reactivity to either one or the other virus. All ELISA reactive samples were assayed at UTEP as described below by a plaque reduction neutralization test (UTEP–PRNT), and at the Arbovirus Laboratory, Wadsworth Center, New York State Department of Health, Slingerlands, New York (NY—PRNT), and by a multiplex microsphere immunoassay (NY—MIA) at the Diagnostic Immunology Laboratory at the Wadsworth Center NY State Department of Health, Albany, NY.

### Indirect immunoglobulin M (IgM) capture ELISA

Cord-blood plasma samples that were reactive for DENV IgG antibody were tested for DENV IgM antibody by an IgM-capture ELISA as previously described [30]. The procedures for the calculation of the OD cut-off value and antibody positive samples and titers were performed and identified as described above for WNV and DENV IgG antibody.

### Plaque reduction neutralization test (PRNT)

The UTEP—PRNT was performed at the UTEP to determine the virus type specific neutralizing antibody that was detected by the ELISA for either DENV or WNV or for both viruses. The WNV PRNT was performed in rhesus monkey (*Macaca mulatto*) epithelial (LLc-MK2) cells using napthol blue black as a stain for viable cells, otherwise the method was similar to the plaque assay described previously that employed Vero cells and crystal violet as the stain [31]. The DENV PRNT used was adapted from the method previously described using BHK-21 Clone 15 cells [32]. A random sub-sample (n = 36) of the plasma samples that were tested for neutralizing antibody at UTEP–PRNT_80_ and the NY-MIA were retested for neutralizing antibody by the NY–PRNT_90_ using the dilution of serum samples that reduced the virus dose by 90% as the antibody titer according to previously described methods [33]. The testing of these 36 samples was to further evaluate the results of the UTEP- PRNT_80_ by an external laboratory using a PRNT_90_ because 90% neutralization provides more robust conclusions than the PRNT_80_. Antibody titers ≥20 were considered positive for virus-neutralizing activity by the NY-PRNT_90_. Samples that neutralized both DENV and WNV and the virus with the antibody titer ≥ 4-fold difference was interpreted as representing the virus type specific antibody with the highest titer.

### Validation of the antibody testing performance of the ELISA and PRNT techniques

A panel of 10 known antibody positive human sera samples to West Nile virus and 10 dengue virus antibody positive human sera samples with titer ranging from 1:10 to 1:640 and 10 antibody negative samples to these viruses were used to verify the results each time the ELISA and plaque reduction neutralization test were performed to test the human plasma sample for antibodies to these viruses. The samples were obtained from an archived serum bank at UTEP, El Paso, Texas and the results of the testing consistently demonstrated that the techniques performed properly.

### Multiplex microsphere immunoassay (MIA)

All DENV and/or WNV ELISA antibody positive plasma samples and all neutralizing antibody positive samples as well as selected antibody negative samples were tested by a multiplex microsphere immunoassay (MIA) at the Diagnostic Immunology Laboratory at the Wadsworth Center, New York State Department of Health (NY-MIA). The NY–MIA was employed to further confirm the identity of virus type specific antibody simultaneously using broadly cross reactive WNV envelope proteins and WNV, ZIKV and DENV virus specific non-structural proteins as antigens. The MIA was performed using a 3 step suspension phase that included 9 recombinant flaviviral non-structural protein antigens to test plasma samples from mothers selected as described above and conducted as described previously [34–36]. The ZIKV envelope (E), ZIKV nonstructural (NS)1, and NS1s of Den 1—Den 4 recombinant protein antigens were purchased from Meridian Bioscience Inc, Cincinnati, OH. The WNV E protein antigen was provided by Michel Ledizet of L Squared Diagnostics (L2DX of New Haven, CT) as described previously [35]. The WNV NS1 was prepared by Randall Renshaw of Cornell University Animal Health Diagnostic Laboratory, Ithaca, NY and WNV NS5 protein antigen was provided by Pei Yong Shi of UTMB, Galveston, TX [34]. Each of these 9 recombinant protein antigens (50ug) were covalently coupled to 6.25 million Luminex MicroPlex microspheres carboxylated polystyrene micro particles (Luminex Corporation, Austin, TX, USA) in accordance to previously published procedures [35] The MFI cutoff values calculated for this survey for ZIKV E and NS1 were 1643 and 452, for WNV E and NS1 and NS5, the cut-of values were 232, 212 and 6680, and for DENV 1–4 serotypes NS 1 antigens, the cut-off values were 808, 746, 615 and 405, respectively. These MFI values were used to interpret antibody positive and negative plasma samples in this survey, with values above the cutoff for only WNV envelope being antibody positive for flavivirus envelope. Samples that had MFI values higher than the cut-off values for WNV envelope and for WNV NS1 and/or NS5 were positive for WNV antibody and samples that had MFI values higher than the cut-off values for WNV envelope and for DENV NS1s for one or more each serotypes were positive for DENV antibody. Samples with MFI values higher than the cut-off values for WNV envelope and for WNV NS1 and/or NS5 and for DENV NS1s for one or more serotypes were considered antibody positive for both WNV and DENV antibodies. The MIA was validated using known antibody negative and positive human sera samples for ZIKV, DENV, and WNV because cross-neutralization reactivity elicited by ZIKV, WNV and DENV *infections* could have been derived from one of the following groups of individuals: (i) infected with ZIKV, WNV and DENV, (ii) infected with ZIKV only but had antibodies cross-reactive to DENV and WNV, (iii) infected with DENV only or multiple infections but had cross-reactive antibodies to ZIKV and WNV, and (iv) infected with WNV only with cross reactive antibodies to both ZIKV and DENV. The inclusion of the different recombinant proteins as antigens in the MIA was to improve the specificity through detection of virus-type specific antibodies. The ZIKV E antigen detected flavivirus antibody, and therefore, ZIKV NS1 antigen was included to detect ZIKV specific antibody. Similarly, WNV E antigen was included to detect flaviviral antibody, and the WNV NS 1 and NS 5 were added to detect WNV specific antibody. In addition, recombinant DENV NS1 proteins from each of the four serotypes were included to detect DENV specific antibody and to differentiate DENV antibodies from those elicited by ZIKV and WNV infection.

## Results

A total of 1,472 mothers were enrolled in this study, including 562 at the Providence Memorial hospital (PMH), 158 at the Sierra Medical Center Main hospital, and 752 at the Sierra Medical East hospital. Overall, 90.8% (n = 1337) of the mothers were from El Paso, Texas, 3.1% (n = 45) from New Mexico, 3.7% (n = 55) from Mexico, and 2.3% (n = 35) of the mothers failed to provide an address. The distribution of the mothers by age revealed that 33.4% (n = 492) of the mother ranged in age from 18–23 years, 38.5% (n = 567) from 24–29, 18.1% (n = 267) from 30–34, 7.0% (n = 103) from 35–40, 1.2% (n = 17) from 41–45, 0.1% (n = 2) was over 46 years old, and 1.6% (n = 24) did not state their age.

Of the 1,472 cord-blood plasma samples obtained from mothers at the time of the delivery of their babies, 4.9% (n = 72) were reactive for DENV IgG antibody by ELISA, suggesting that the mothers had been infected with one or more of the 4 DENV serotypes. However, since reactivity for DENV IgG antibody could represent cross reactivity due to a previous WNV infection, the samples were further tested for WNV ELISA IgG antibody. The results indicated that all but 2 of the 72 samples were also reactive for WNV IgG antibody, thus reflecting infection by either one or both viruses for 70 samples. All reactive samples tested by ELISA at 2 –fold dilutions ranging from 1:100 to 1:6400 for DENV and WNV IgG antibodies were reactive at all titers for both viruses. The results of testing the 72 ELISA reactive samples by the UTEP- PRNT and NY- MIA for both DENV and WNV antibodies revealed that 59 were positive to either DENV or WNV or to both viruses, and/or for antibody to flavivirus envelope. The other 13 ELISA reactive samples or 18% (13/72) were negative for DENV and/or WNV antibodies or to both viruses by the UTEP- PRNT and NY- MIA. An additional 28 samples negative for DENV and WNV ELISA IgG antibodies were selected for testing by the UTEP-PRNT and the NY-MIA to evaluate the validity of the ELISA results. All 28 samples were negative for antibody by both assays. Also, among the 59 antibody positive samples by the ELISA and the UTEP-PRNT and NY- MIA, 36 were selected for further testing by the NY—PRNT_90_ to further evaluate the validity of the results obtained by the ELISA, UTEP–PRNT_80_ and the NY—MIA. The results showed that all but 2 samples were in concordance for the different assays. A more detailed description of these observations are described below.

Of the 59 samples that were positive for either DENV and/or WNV antibodies, 3.0% (45/1,472) were positive for only WNV antibody as evidence of a previous infection (Table 1). The distribution of the WNV antibody positive samples included 1.8% (10/562) of mothers enrolled at the PMH hospital, 4.4% (7/158) at the SMC hospital and 3.7% (28/752) at the SES hospital. Antibody to only DENV was detected in 0.5% (8/1,472) of the mothers as evidence of a previous infection by this virus. The distribution of DENV antibody included 0.0% (0/562) among mothers enrolled at the PMH hospital, 1.3% (2/158) at the SMC hospital, 0.8% (6/752) at the SES hospital. Also, antibodies to both DENV and WNV were detected in one mother at the PMH hospital and 2 mothers at the SES hospital. These 3 mothers with evidence of a previous WNV/DENV infection increased the overall WNV antibody prevalence rate to 3.3% (48/1,472), and to an overall rate of 0.7% (11/1,472) for DENV antibody. Another 5 mothers had antibody only to flavivirus envelope by the NY-MIA, but 4 of these the mothers were positive by the UTEP-PRNT for WNV antibody, and one for WNV/DENV antibody. Also, 3 mothers, 2 at the SMC and one at the SES hospitals had antibody to flavivirus envelope by the NY-MIA but were negative for DENV and/or WNV antibodies. The overall seroprevalence rates for flaviviruses for mothers at the different hospitals was 4.0% (59/1,472), including 2.1% (12/562) at the PMH, 7.0% (11/158), and 4.8% (36/752) at the SES. All 59 samples tested by the NY—MIA were negative for ZIKV antibody.

**Table 1:** Seroprevalence rate for DENV and WNV antibody in 59 cord blood samples from mothers at the time of delivery of newborns in selected El Paso, Texas hospitals.

Antibodies	Providence Memorial Hospital [no. (%)]	Sierra Medical Center Main Hospital	Sierra Medical East Hospital	Seroprevalence Rate
**West Nile**	10 (1.8%)	7 (4.4%)	28 (3.7%)	45 (3.0%) *
**Dengue**	0	2 (1.3%)	6 (0.8%)	8 (0.5%) **
**Dengue/West Nile**	2 (0.4%)	0	1 (0.1%)	3 (0.2%)
**Flavivirus Envelope only**	0	2 (1.3%)***	1 (0.1) ***	3 (0.2%)
**Total**	12/562 (2.1%)	11/158 (7.0%)	36/572 (4.8%)	59/1,472(4.0%)

*- three additional samples positive for antibody to both DENV and WNV were added to the 45 WNV antibody positive mothers to equal 48 or 3.3% of the total samples tested

**- three additional samples positive for antibody to both DENV and WNV were added to the 8 DENV antibody positive mothers to equal 11 or 0.75% of the total samples tested.

***-seven samples were positive for antibody to flavivirus by the MIA, 4 of which were positive for WNV antibody and 3 were negative by the PRNT for both WNV and DENV antibodies.

Among the 11 of 1,472 mothers who had DENV antibody, 6 had not traveled outside of El Paso, Texas during the past 6 months, but one had traveled to Jamaica and one to Puerto Rico. The 3 mothers who were positive for both DENV and WNV antibodies had traveled to Mexico. Of the 45 of 1,472 mothers who had WNV antibody, 22 had not traveled outside of El Paso, 18 had traveled to Mexico, one to Puerto Rico, one to Europe, one to Costa Rico, one to Europe, Asia and Caribbean and one did not provide an answer.

The results of testing 12 ELISA WNV and DENV antibody positive samples obtained from 562 mothers at the PMH hospital by the UTEP-PRNT and NY–MIA are presented in S1 Table. Among these samples, 10 were positive for WNV antibody and 2 were positive for both WNV and DENV virus by the UTEP-PRNT. The results for the NY-MIA included 8 with antibody to WNV, 3 with antibody to both WNV and DENV and one with only flavivirus antibody. None of the samples were positive for DENV antibody only. Eight of WNV and 2 WNV/DENV antibody positive samples were in agreement between the UTEP-PRNT and NY- MIA. The results for the other 2 samples were discordant, including sample PMH0037 that was positive for antibody to flavivirus envelope by the NY- MIA, but positive for WNV antibody by the UTEP- PRNT (Table 2). The other sample, or PMH0167 was positive for DENV/WNV antibody by the NY- MIA, but positive for only WNV antibody by the UTEP- PRNT. The ELISA IgG antibody titers for 11 samples were 4- fold greater for WNV antibody. The other sample was one of the 2 WNV/DENV antibody positives that had the same ELISA antibody titer for WNV and DENV.

**Table 2:** Summary of disconcordant West Nile and dengue virus neutralizing antibody results between the New York multiplex microsphere assay (NY—MIA) and the University of Texas at El Paso plaque reduction neutralization test (UTEP—PRNT) for 9 of 59 cord-blood plasma samples obtained from mothers at the Providence, Sierra East, Sierra Main hospitals, El Paso, Texas.

			Median Fluorescence Intensity Values of samples reactive with West Nile virus envelope and/or West Nile and dengue virus (DEN) nonstructural antigens								UTEP PRNT_80_ Titers		
	ELISA IgG Antibodies		WNV-E	WNV-NS1	WNV-NS5	Den 1 NS1	Den 2 NS1	Den 3 NS1	Den 4 NS1	NY—MIA	Antibody Titers		PRNT_80_
Sample Code	DENV	WNV	232*	212	6680	808	746	615	405	Diagnosis antibody	DENV	WNV	Diagnosis antibody
PMH0037	400	1600	1004	171	459	14	10	41.5	17	Flavivirus Envelope	≤20	320	WNV
PMH0167	1600	6400	5523	1048	1300	2498	2630	8207	2944	WNV/DENV	≤20	40	WNV
SES0146	400	100	737	262	2862	488	382	860	345	Flavivirus Envelope	≤20	≤20	Neg
SES0450	400	6400	364	81	629	19	29	20	19	Flavivirus Envelope	≤20	1280	WNV
SES0632	400	6400	620	179	504	19	15	62	31	Flavivirus Envelope	≤20	1280	WNV
SES0654	1600	6400	2343	249	1053	46	53	55	46	WNV	DENV3 (160), DENV4 (640)	WNV (320)	WNV/DENV
SES0827	1600	1600	273	53	658	273	113	461	78	Flavivirus Envelope	≤20	320	WNV
SMC0055	1600	1600	407	30	430	12	10	107	15	Flavivirus Envelope	≤20	≤20	Neg
SMC0119	200	≤100	851	33	331	564	149	310	71	Flavivirus Envelope	≤20	≤20	Neg

*- Median fluorescence intensity cut-off values for test samples equal to or higher representing antigen–antibody reactivity, ELISA cut-off values DENV IgG cut off = 0.24–0.29, WNV IgG cut off = 0.11–0.16, samples positive for both DENV and WNV antibodies with the same antibody titers or less than 4-fold difference = antibody positive to both viruses, virus with 4-fold or greater antibody titer considered antibody positive for the virus with the highest antibody titer. (7 flavivirus and 2 WN and/or DEN)

The results for screening of 36 ELISA WNV and DENV antibody positive samples obtained from 752 mothers at the SES hospitals by the UTEP-PRNT and NY–MIA are presented in S2 Table. Of the 36 reactive samples, 28 were positive for WNV, 6 for DENV, and one for WNV/DENV antibodies by the UTEP-PRNT. The results for the NY-MIA revealed that 26 of the 36 samples were positive for WNV antibody, 4 were positive for flavivirus envelope antibody and 6 were positive for DENV antibody by the NY- MIA. Three of the 4 flavivirus antibody positive samples were positive by the UTEP- PRNT for WNV antibody, and one flavivirus envelope antibody positive was negative to both DENV and WNV by the UTEP-PRNT. The results for 31 SES samples were in concordance between the NY- MIA and the UTEP—PRNT and the results for 5 samples were discordant (S2 Table). Four of the 5 discordant samples, included samples, SES0450, SES0827, SES0654 and SES0632 that were positive for flavivirus envelope antibody by the NY—MIA, but 3 were positive for antibody to WNV and one for antibody to WNV/DENV by the UTEP–PRNT (Table 2). The other sample SES0146 was positive for antibody to flavivirus envelope and negative for antibody by the UTEP-PRNT. The ELISA antibody titers for 19 samples were 4-fold greater to WNV than to DENV, 8 with antibody titers equal to both DENV and WNV viruses, and one with a titer two-fold higher for WNV. The ELISA IgG antibody titers for the 6 DENV confirmed antibody positive mothers enrolled at the SES hospital ranged from 1:1600 to 1:6400, with titers for 4 samples 4-fold higher to DENV than for WNV, and 2 samples had antibody titers equal to both DENV and WNV.

The results for screening of plasma samples for 158 mothers enrolled at the SMC hospital by ELISA for DENV and WNV IgG antibodies and for UTEP-PRNT and NY-MIA are presented in S3 Table. Of the 11 ELISA reactive samples, 7 were positive for WNV antibody by the UTEP-PRNT and/or NY-MIA and all had titers 4-fold greater to WNV than to DENV. Of the 2 DENV antibody positive samples, one had a titer equal to both viruses and one had a titer 4 fold higher for DENV than WNV. Two sample were positive only for flavivirus envelope antibody, one with an IgG antibody titer equal for DENV and WNV, and one with a 1:200 titer to DENV and negative for WNV antibody. The results for 9 samples were in concordance between the NY-MIA and the UTEP- PRNT and discordant for 2 samples (Table 2). The 2 samples, including SMC0055 and SMC0119 were positive for antibody to flavivirus envelope by the NY—MIA, but were negative for antibody by the UTEP- PRNT.

A summary of the 9 samples that yielded discordant results is presented in Table 2. Of the 9 samples, 7 were positive for antibody to the flavivirus envelope by the NY–MIA, one was positive for WNV/DENV antibody, and one was positive for WNV antibody alone. In contrast, the UTEP-PRNT showed that of the 7 samples positive for flavivirus envelope antibody, 4 were positive for WNV antibody, and 3 were negative. The one NY—MIA WNV/DENV antibody positive sample was positive by the UTEP–PRNT for WNV antibody and the one NY—MIA positive for only WNV antibody, was positive for antibody to WNV and DENV by the UTEP–PRNT. None of these samples were tested by the NY-PRNT.

As mentioned above, 13 samples were reactive for ELISA IgG antibody to either or both WNV and DENV, but negative by NY- MIA and UTEP- PRNT (Table 3). Of these 13 samples, 3 were collected from mothers at the PMH, 9 at SES and one at the SMC hospital. All were positive for DENV antibody by the ELISA with 11 having a titer of 1:400, one had a titer of 1:200, and one had a 1:100 titer. Only 5 of the 13 DENV reactive samples were reactive to WNV, including one with a titer of 1:400 and 4 with titers of 1:100. Overall, 13 samples were reactive to DENV, and 5 of the 13 were also reactive to WNV.

**Table 3:** Summary of 13 plasma samples obtained from mothers at the Providence, Sierra East, Sierra Main hospitals, El Paso, Texas that were positive for West Nile and dengue virus antibody by the ELISA, but negative by the multiple microsphere assay (MIA) and the UTEP plaque reduction neutralization test (PRNT).

			Median Fluorescence Intensity Values of samples reactive with West Nile virus envelope and to West Nile and dengue virus nonstructural antigens								UTEP PRNT_80_ Titers		
	ELISA IgG Antibodies		WNV-E	WNV-NS1	WNV-NS5	Den 1 NS1	Den 2 NS1	Den 3 NS1	Den 4 NS1	MIA	Antibody Titers		UTEP PRNT_80_
Sample Code	DEN	WN	232	212	6680	808	746	615	405	Diagnosis antibody	DENV	WNV	Diagnosis antibody
PMH0034	400	≤100	194	199	790	115	93	461	162	Neg	≤20	≤20	Neg
PMH0105	400	≤100	18	51	424.5	11	12	61	17	Neg	≤20	≤20	Neg
PMH0124	400	400	300	56	1293	90	150	439	26	Neg	≤20	≤20	Neg
SES0061	100	100	4	34	249	8	6	22	13	Neg	≤20	≤20	Neg
SES0204	400	≤100	11	55	559	25	181	35	15	Neg	≤20	≤20	Neg
SES0214	400	≤100	11	69	1158	11	18	38	19	Neg	≤20	≤20	Neg
SES0247	200	≤100	16	49	399	28	16	47	16	Neg	≤20	<20	Neg
SES0279	400	100	76	71	561	39	24	180	51	Neg	≤20	≤20	Neg
SES0282	400	≤100	17	56	1148	21	14	49	26	Neg	≤20	≤20	Neg
SES0284	400	≤100	8	32	292	6	6	48	12	Neg	≤20	≤20	Neg
SES0451	400	100	295	91	921	52	36	227	68	Neg	≤20	≤20	Neg
SES0804	400	≤100	141	84	939	251	66	228	219	Neg	≤20	≤20	Neg
SMC0017	400	100	10	46	1526	33	27	29	17	Neg	≤20	≤20	Neg

Median fluorescence intensity cut-off values for test samples equal to or higher representing antigen–antibody reactivity, ELISA cut-off values DENV IgG cut off = 0.24–0.29, WNV IgG cut off = 0.11–0.16, samples positive for both DENV and WNV antibodies with the same antibody titers or less than 4-fold difference = antibody positive to both viruses, virus with 4-fold or greater antibody titer considered antibody positive for the virus with the highest antibody titer.

An additional 28 non-reactive samples by the ELISA were selected, including 3 collected from mothers at the PMH hospital, 18 at the SES and 7 at the SMC for testing by the NY- MIA and UTEP- PRNT to evaluate the accuracy of the ELISA (S4 Table). The 28 samples were negative for DENV and WNV antibody by the NY- MIA and UTEP- PRNT, thus confirming that the ELISA antibody negative results were in concordance with the NY—MIA and UTEP—PRNT negative results.

A subsample of 36 of the 72 plasma samples, including 15 antibody negative samples and 14 WNV antibody and 6 DENV antibody and one WNV/DENV antibody positive samples were randomly selected for further testing by a NY- PRNT using a 90% reduction of the virus dose (Table 4). The rationale for testing samples by the NY-PRNT was to have an external laboratory test selected samples to further evaluate the NY- MIA and UTEP—PRNT results as an enhanced robust testing scheme. All 36 samples were concordant except for 2 by the 3 assays. The 2 discordant samples included sample SES0475 that was positive for WNV/DENV by the NY- PRNT, and positive only for WNV by the NY- MIA and UTEP—PRNT. The other sample, SES0654 was positive for WNV antibody only by the NY-PRNT, but positive for both WNV/DENV antibody by the UTEP- PRNT and positive for antibody to flavivirus envelope by the NY- MIA.

**Table 4:** Summary of DENV and WNV antibody results for 36 cord blood samples obtained from mothers at the time of delivery of newborns in the Providence Memorial, Sierra East, and Sierra Main hospitals, El Paso, Texas. Samples were assayed by the New York plaque reduction neutralization test (NY—PRNT) and had previously been tested by the New York multiplex microsphere assay (NY-MIA) and the University of Texas at El Paso plaque reduction neutralization test UTEP—PRNT, including 13 WNV and 7 DENV antibody positive and 16 antibody negative samples.

	NY- PRNT_90_					UTEP PRNT_80_	NY- MIA
Sample Code	DENV-2 antibody screen	DENV-2 antibody titer	WNV antibody screen	WNV antibody titer	Diagnosis antibody	Diagnosis antibody	Diagnosis antibody
**PMH0014**	Neg	<20	NEG	<20	Neg	Neg	Neg
**PMH0034**	Neg	<20	NEG	<20	Neg	Neg	Neg
**PMH0105**	Neg	<20	NEG	<20	Neg	Neg	Neg
**PMH0124**	Neg	<20	NEG	<20	Neg	Neg	Neg
**PMH0149**	Positive (Pos)	20	POS	1280	WNV/DENV	WNV/DENV 2	WNV/DENV
**PMH0247**	NEG	<20	NEG	<20	Neg	Neg	Neg
**PMH0279**	Neg	<20	POS	320	WNV	WNV	WNV
**SES0031**	Neg	<20	POS	80	WNV	WNV	WNV
**SES0045**	Neg	<20	POS	320	WNV	WNV	WNV
**SES0059**	Neg	<20	POS	80	WNV	WNV	WNV
**SES0068**	Pos	160	Neg	<20	DENV	DENV-2	DENV
**SES0073A**	Neg	<20	POS	640	WNV	WNV	WNV
**SES0214**	Neg	<20	Neg	<20	Neg	Neg	Neg
**SES0228**	Neg	<20	Neg	<20	Neg	Neg	Neg
**SES0248**	Pos	160	Neg	<20	DENV	DENV 2	DENV
**SES0252**	Neg	<20	Neg	<20	Neg	Neg	Neg
**SES0264**	Neg	<20	Neg	<20	Neg	Neg	Neg
**SES0286**	Neg	<20	Neg	<20	Neg	Neg	Neg
**SES0288**	Neg	<20	Neg	<20	Neg	Neg	Neg
**SES0402**	Neg	<20	Neg	<20	Neg	Neg	Neg
**SES0405**	Neg	<20	Neg	<20	Neg	Neg	Neg
**SES0447**	Pos	80	Neg	<20	DENV	DENV1 & 2	DENV
**SES0475**	Pos	20	Pos	320	WNV/DENV	WNV	WNV
***SES0500***	Pos	80	Neg	<20	DENV	DENV-2	DENV
**SES0654**	Neg	<20	Pos	320	WNV	WNV/DENV	WNV
**SES0664**	Neg	<20	Pos	320	WNV	WNV	WNV
**SES0669**	Neg	<20	Pos	80	WNV	WNV	WNV
**SES0670**	Neg	<20	Pos	160	WNV	WNV	WNV
**SMC0008**	Neg	<20	Pos	320	WNV	WNV	WNV
**SMC0040**	Neg	<20	Pos	640	WNV	WNV	WNV
**SMC0041**	Neg	<20	Neg	<20	Neg	Neg	Neg
**SMC0067**	Pos	80	Neg	<20	DENV	DENV-2	DENV
**SMC0068**	Neg	<20	Pos	640	WNV	WNV	WNV
**SMC0070**	Neg	<20	Pos	80	WNV	WNV	WNV
**SMC0114**	Pos	320	Neg	<20	DENV	DENV-1	DENV
**SMC0451**	Neg	<20	Neg	<20	Neg	Neg	Neg

Overall, the results for the UTEP-PRNT and the NY-MIA were in concordance for 84.7% (50/59) of the DENV and/or WNV antibody positive samples. The 13 ELISA reactive samples were negative by both the UTEP–PRNT and the NY-MIA, and the 28 ELISA negative samples were negative by both the NY-MIA and UTEP- PRNT. Therefore, for 100 samples tested, the total concordance between the UTEP-PRNT and the NY-MIA was 91.0% (91/100). Among 36 samples selected, including 13 WNV and 7 DENV antibody positive samples and 16 negative samples by the UTEP-PRNT and the NY-MIA, the results of testing these samples by the NY–PRNT were in concordance for 94.4% (34/36) of the samples. Overall, the diagnostic results, obtained by the 3 different tests together provided conclusive evidence of the estimates of the frequency of maternal transfer of DENV and WNV specific antibodies among child-bearing mothers in the El Paso community.

## Discussion

Surveillance for human DENV infection along the U.S.—MX border has been conducted primarily during or after sporadic outbreaks of dengue involving endemic transmission in selected southern urban communities in the Rio Grande valley of TX. Seroprevalence rates as evidence of past DENV infection ranged from 23% in Laredo, TX to 38% in Brownsville, TX [8, 37]. More recently, observations indicated that during 2015, 14.1% of a selected cohort of 77 adults had serological evidence of a past DENV infection and 10.2% (5/61) seroconverted as evidence of a recent DENV 1 and 2 infection the El Paso adjoining sister community of Ciudad Juarez, MX [12]. Other than the Rio Grande valley region of TX, autochthonous transmission of DENV has not been confirmed for any other U.S. border communities [13]. A possible explanation for the perceived absence of DENV in other urban border communities is that cases may not have been detected by passive surveillance, or the lack of surveillance and therefore, this survey was conducted to determine if these viruses were endemic in the El Paso, TX community. The findings revealed that 0.74% (11/1,472) of cord blood plasma samples obtained from mothers at the PMH, SES and SMC hospitals in El Paso were positive for DENV 1 and/or 2 IgG antibody and 3.3% (48/1,472) were positive for WNV antibody. The identity of these virus-type specific antibody was confirmed by a robust testing scheme that involved the screening of samples by an ELISA and by further analyzing the ELISA reactive samples by the more specific assays, including the NY—MIA and the UTEP—PRNT_80_ and the NY—PRNT_90_. The use of these different diagnostic tests was employed because the serological diagnosis of virus type specific flavivirus antibody is often confounded by the antigenic similarities among the viruses of the F*lavivirus* genus [38, 39].

The validity of our serological findings was supported by previous reports that showed the assays employed in this survey to be appropriate for detecting and distinguishing among antibody elicited by human flavirvirus infections.(29 tardi, 30 innnis, 32 moren, 33 lindsey, 34 and 35 wong). The result of this survey showed that the sensitivity of the ELISA IgG antibody technique and the PRNT was comparable for detecting WNV and DENV antibodies. However, the lack of specificity of the ELISA IgG antibody screening technique as used in this study detected IgG antibody to one or both DENV and WNV, thus, detecting antibody reactive to the broadly cross reacting envelope E antigen that is common to all Flaviviruses [29, 30, 38, 39]. As a result, the ELISA reactive plasma samples were tested by the more specific PRNT technique. The PRNT results confirmed the diagnosis of virus type specific WNV and DENV antibodies in this survey and was consistent with reported observations that the PRNT is among the most sensitive and specific serologic test available for detecting virus type specific flavivirus antibody [32–34] As a supplement to the PRNT, the MIA was performed using flavivirus structural envelope protein(s) as antigens in combination with virus type specific non-structural protein antigens that provided results comparable to the PRNT for detecting virus type specific flavivirus antibody [32–34]. These results further confirmed that the MIA offers an alternative and reliable technique to the laborious and time-demanding PRNT for detecting virus type specific flavivirus neutralizing antibodies. In addition to the latter observations, the confirmation of the UTEP serological results by PRNT and the MIA testing technique in an external laboratory, or at the Wadsworth Center, New York State Department of Health further supports the validity of the serological results of this survey”.

Among the 11 DENV antibody positive cord-blood plasma samples, 2 were obtained from mothers enrolled at the PMH and one from a mother enrolled at the SES hospital; all 3 were positive for antibody to both DENV and WNV. The other 8 DENV antibody positive mothers included 7 enrolled at the SES and 2 at the SMC hospital. Of the 11 antibody positive mothers, 8 resided in East El Paso, 2 in Central and one in West, El Paso. Most of the DENV antibody positive mothers from East El Paso were in the area of the city where the population density of *Aedes aegypti* was the highest during the summer and fall seasons from 2015–2017 with estimates of 205 females per month for at a total of 3,768 female mosquitoes. (Watts D.M., unpublished data). Also, as a potential vector of DENV, data accumulated from 2015–2017 indicated that this species was distributed throughout the El Paso community and was second to *Culex quinquefasctiatus* as the most abundant species. Further suggestive evidence of the possibility that the mothers may have acquired a local DENV infection was supported by the travel history. Of the 11 mothers, 6 had not traveled outside of El Paso, Texas, and 3 had traveled to Mexico, one to Puerto Rico, and one had traveled, but not to a DENV endemic country. These observations and the more recent evidence that supported endemic transmission among a human cohort with DENV infection in Ciudad Juarez, MX, the urban adjoining sister community with El Paso suggested that the mothers could have acquired DENV infections in the El Paso community [12].

Although DENV infection in the El Paso community cannot be excluded, the location and date of infection are inconclusive. Blood samples were not obtained from the mothers; therefore, it was not possible to test for IgM antibody as possible evidence of a recent DENV infection. Also, our results that showed the 11 DENV IgG antibody positive cord-blood samples were negative for IgM antibody was consistent with general knowledge that IgM antibody rarely crosses the placental barrier [40]. However, DENV IgG antibody has been well documented to be transferred across the placental barriers in several DENV endemic countries. In support of these observations, the results of several studies documented that DENV IgM antibody was rarely or not maternally transferred at all, but that IgG antibody was transferred at a frequency of 90% or higher to neonates in Thailand, Brazil, and Malaysia [41–46]. Therefore, these results suggested that antibody rates in cord-blood samples may provide a reliable estimate of the prevalence of DENV infection in the female population for child bearing age cohort, but may or may not reflect the rate among the same male cohort. Estimates of the distribution of cases by gender in 6 countries in Southeast Asia found a consistent and significant excess of dengue cases among males ≥ 15 years of age [24]. However, studies in South and Central America found that dengue cases were equally distributed among male and female, or a greater proportion of female cases [25–27]. Also, in the US-Mexico border community of Reynoso, MX, 58% of dengue cases during an outbreak between 2014–2016 were among females [47]. These findings represented observations in DENV endemic countries, and therefore, the relevance, if any to the prevalence rate and the gender distribution of DENV antibody in the El Paso community will depend on whether or not the El Paso results reflected endemic DENV transmission.

The frequency of DENV IgG antibody that transferred across the placental barriers may or may not be representative of the prevalence of DENV infection in the mothers, or the El Paso community at large as possible evidence of endemic transmission. However, the exceptionally low DENV antibody prevalence rate of 0.74% in El Paso in comparison to much higher rates in well documented DENV endemic areas argue against endemic transmission. For example, the DENV seroprevalence rate was 38% in Brownsville, TX and 77% Matamoros, MX, 23% in Laredo, TX, and 48% in Nuevo Laredo, MX, and 62.7% in Reynoso, MX [8, 15, 47]. The DENV seroprevalence based on a regional analysis among all age groups and adults (>15 years old) involving a sample size that varied from 46 to 5,669 was 64.4% in the Americas, 46.2% in Asia and 18.1% in Africa [48]. The rates in other selected DENV endemic urban communities varied, for example, the seroprevalence rate for DENV ranged from 61% to 97% in Zhejiang Province, China, Pune, India, Central and Southern Thailand, Medellin, Colombia, Bangkok, Thailand, Managua, Nicaragua, and Iquitos, Peru [49–55]. Overall, these higher DENV seroprevalence rates in well documented DENV endemic regions suggested that the exceptionally low prevalence rate of DENV antibody in the El Paso community did not reflect widespread autochthonous transmission of DENV.

Active surveillance for human cases of dengue has not been conducted, and cases have not been reported based on passive surveillance in the El Paso community. Also, as further evidence that argues against endemicity of DENV, these viruses were not isolated during 2015–2017 from a total of 3,768 female *Aedes aegypti* collected in East El Paso where DENV antibody was detected in 7 of the 11 DENV antibody positive mothers enrolled in this survey (Watts D.M., unpublished data). Although the reported DENV infection rates in endemic countries varied for *Ae*. *aegypti*, estimates indicated that the population density and number tested in El Paso were likely to be sufficient to detect DENV, if these viruses were endemic in this community. Estimates of minimum field DENV infection rates (MFIR) in *Ae*. *aegypti* based on the testing of a 1000 mosquitoes in a DENV hyper-endemic urban community of Bangkok Thailand varied from 0.4 to 8.2 isolates per 1000 female mosquitoes around houses with positive dengue cases and 0.4 per 1000 near houses with no dengue cases [56]. In other DENV endemic countries, the MFIR/1000 mosquitoes ranged from 0.15 to 26.0 in Puerto Rico, 0.5 to 32 in Singapore, 5.8 in New Delhi, India, 16.2 in Brazil, 15.9 in Venezuela, 18.0 in MX and the rate in Colombia was 11.7 mosquitoes during an epidemic and decreased to 3.56 after the epidemic [57–63].

As an alternative explanation for the serological evidence of a very low prevalence of previous DENV infections in the El Paso community is the possibility that imported cases of DENV were responsible for focal and transient transmission that resulted in a few autochthonous cases. The reported occurrence of all of the sporadic outbreaks of DENV involving one to 26 autochthonous cases from 1980 to 2013 in Brownsville, TX and surrounding communities were associated with DENV infected travelers returning from visits to the bordering Mexican city of Matamoros during epidemics [9, 10, 37, 64]. The implementation of surveillance in Southern France and Croatia revealed that two autochthonous cases of dengue occurred during 2010 in each of these countries and originated from imported cases by viremic travelers [65]. Thus, the very low number of secondary autochthonous cases that were acquired from viremic travelers may go undetected especially in areas without active surveillance programs and because asymptomatic or silent DENV infections are much more frequent and more difficult to detect than symptomatic cases [66].

The growing emergence and geographical co-endemicity of different flaviviruses has further complicated diagnostic and research serological testing results by antibody cross-reactivity among the flaviviruses and the original antigenic sin phenomenon [29, 38, 67, 68]. The underlying reason is because the flavivirus humoral antibody response is predominantly generated against the highly immunogenic envelope protein that contains both flavivirus cross-reactive epitopes and virus-specific epitopes [69, 70]. Also, individuals who are sequentially infected by a heterologous flavivirus experience a boost in antibody titer against the original virus, thus resulting in a phenomenon known as 'original antigenic sin’. As a result, the heterologous flavivirus infection elicits a greater serological response to the first infecting virus and therefore can result in false serological diagnoses. The multiple, complex epitope composition of flaviviruses and the associated immune response to the epitopes has presented technological challenges, especially to overcome antibody cross-reactivity complicating interpretation of the typical antibody screening methods such as ELISA and immunofluorescence assay [71, 72]. While the neutralization test continues to be the “gold standard” as the most accurate serologic test available for flavivirus diagnosis, this procedure has been supplemented by the identification and use of flavivirus structural envelope protein(s) in combination with virus type specific non-structural proteins to develop immunoassay [33, 34, 73]. As an example, the combinations of these proteins as antigens were used to develop a Multiplex Microsphere Immunoassay [34, 74]. This assay is more rapid and comparable in sensitivity and specificity to the more laborious, time demanding and BSL 3 containment requirements of the neutralization assay for characterizing and quantifying circulating levels of flavivirus neutralizing antibody. The complications involved in making an accurate serological diagnosis was the overarching rationale in this survey to employ multiple serological assays to obtain a reliable understanding of whether or not DENV were endemic in the El Paso community.

Thirteen of the 72 (18%) plasma sample were positive by the ELISA for DENV and/or WNV IgG antibody, but negative for antibody by the NY—MIA and UTEP—PRNT. All 13 samples were reactive to DENV, including 11 with a titer of 1:400 and one with a titer of 1:200 and one with a 1:100 titer. Only 5 of these samples were reactive to WNV, including one with a 1:400 titer and 4 with a 1:100 titer. An explanation for this pattern of reactivity is unknown, but based on the suggested results of a study in Israel, the reactivity primarily to DENV could be due to the greater sensitivity of the ELISA, or cross reactivity to another known or unknown flavivirus or non-specific reactivity [75]. A study in Kenya showed that of 830 DENV ELISA IgG antibody positive samples that were tested for neutralizing activity, 488 (58.8%) neutralized DENV and 94 (11.3%) neutralized WNV [76]. The remaining 30% (n = 248) of ELISA reactive samples that were negative for DENV and WNV neutralizing antibody were attributed to cross-reacting antibodies elicited by other flaviviruses, or to non-specific reactivity. In this serosurvey, the samples were only tested for DENV and WNV antibodies and therefore, does not exclude the possibility that the low reactivity of the 13 samples to DENV did not reflect cross reactivity to other flaviviruses. The most likely possibility for cross reactivity to another flavivirus would be that some of the mothers were infected with the antigenically closely related St Louis encephalitis [77]. This virus has been isolated repeatedly from *Culex* species mosquitoes in the El Paso community, but has not been reported as a cause of human infections (Texas Department of State Health Services, unpublished data,). Also, that the low reacting samples to DENV may have reflected an unknown cross reacting flavivirus was supported by our results that showed 2 samples with ELISA titer of 1:200 and 1: 400 only to DENV to be confirmed positive by the NY-MIA for antibody to only flavivirus envelope.

As observed in this survey, all but 2 of the 72 samples that were reactive by ELISA were reactive to both WNV and DENV antigens. Therefore, the UTEP–PRNT and the NY—MIA were employed to distinguish WNV from DENV antibody among the ELISA reactive samples. The results of these 2 assays together indicated that 59 samples were positive for flavivirus antibody, including 45 samples that were positive for WNV antibody, 8 were positive for DENV antibody, and that 3 samples were positive for both DENV and WNV antibodies and 3 samples were positive only for antibody to flavivirus envelope. The results for 50 of the 59 samples were in concordance with the NY-MIA and the UTEP PRNT. Of the 9 sample with discordant results, 4 samples were positive by the NY-MIA for only flavivirus envelope antibody, but positive by the UTEP–PRNT for only WNV, suggesting that the latter test was more sensitive for identifying virus type specific antibody. Also, one sample that was positive for both WNV and DENV antibody by the NY–MIA was only positive by the UTEP PRNT for WNV antibody. Another sample was positive for WNV by the MIA was positive for WNV by the UTEP-PRNT for WNV and DENV. In contrast, the NY–MIA detected antibody to flavivirus envelope for 3 samples that were negative by the UTEP–PRNT, suggesting that the samples were positive for antibody to another flavivirus, possibly St. Louis encephalitis virus. An additional 28 ELISA antibody negative samples were also negative for antibody by the NY- MIA and the UTEP–PRNT, thus supporting the accuracy of the ELISA results.

Although 271 human cases of WN were reported annually between 2003 and 2016 in El Paso Texas, serosurveys have not been conducted to determine estimates of WNV IgG antibody as evidence of past infections in the population [19, 78]. Our serosurvey provided further evidence of WNV human infection in the El Paso community, but was focused on rates among child-bearing age females. The 3.3% WNV seroprevalence rate observed in this survey was comparable to the 4% rate reported for 549 cord blood samples obtained from mothers in Colorado [79]. Whether or not seroprevalence rate based on the detection of antibody in cord-blood samples can be interpreted as reflecting the infection rate in the general population is unknown. However, our findings were comparable to the low prevalence of 5.1% among a cohort of adults in Ciudad Juarez, MX but higher than 0.4% symptomatic and 0.25% asymptomatic individuals reported for 899 blood donors in Northern Mexico [12, 80]. The rate in other WNV selected endemic areas ranged from 2.6% to 14% in North America and Canada [81–84]. In Europe and the Middle East, the rates ranged from 2.1% to 11.1%, and from 2.75% to 59% in Africa [76, 85–92]. Although seroprevalence rates varied, the observation of a 3.3% rate based on a sub-samples of cord blood samples in the El Paso community was comparable to the low rates in WNV enzootic areas of North America and Europe, but lower than for most African countries.

In conclusion, our serosurvey was the first to be conducted in the El Paso community for serological evidence of DENV infection. Although the survey showed that 72 of 1,472 human plasma samples were reactive for DENV, the more specific serological tests demonstrated that only 11 samples were positive for DENV antibody and 48 were positive for WNV antibody. While the findings were based on the detection of antibody in cord-blood samples, the seroprevalence rate for WNV was consistent with the rates reported from other WNV enzootic areas and regions. The very low seroprevalence rate for DENV was inconsistent with much higher rates reported for DENV in endemic countries, and therefore suggested that this evidence reflected a transient foci of endemic transmission of the virus in the El Paso community. Furthermore, the reported endemic transmission of DENV in the adjoining sister urban community of Ciudad Juarez, MX and the possibility that the antibody rate using cord-blood samples was not representative of the population warrant further studies to obtain more conclusive understanding of whether or not DENV is endemic in the El Paso community.

## Supporting information

S1 Table Summary of West Nile and dengue virus antibody detected by Enzyme-Linked Immunoassay (ELISA), New York multiplex Microsphere Assay (NY—MIA) and the University of Texas at El Paso Plaque Reduction Neutralization Test (UTEP—PRNT) in 12 plasma samples obtained from 562 mothers at the time of delivery of newborns in the Providence Hospital, El Paso, Texas. (DOCX)

S2 Table Summary of West Nile and dengue virus antibody detected by Enzyme-Linked Immunoassay (ELISA), New York multiplex Microsphere Assay (NY—MIA) and the University of Texas at El Paso Plaque Reduction Neutralization Test (UTEP—PRNT) in 36 plasma samples obtained from mothers at the time of delivery of newborns in the Sierra East Hospital, El Paso, Texas. (DOCX)

S3 Table Summary of West Nile and dengue virus antibody detected by Enzyme-Linked Immunoassay (ELISA), New York multiplex Microsphere Assay (NY—MIA) and the University of Texas at El Paso Plaque Reduction Neutralization Test (UTEP—PRNT) in 11 plasma samples obtained from 158 mothers at the time of delivery of newborns in the Sierra Main Hospital, El Paso, Texas. (DOCX)

S4 Table Summary of 28 plasma samples, including 3 from mothers at the Providence Memorial, 18 from mothers at the Sierra East, and 7 from mothers a the Sierra Main hospitals, El Paso, Texas that were negative for antibody by ELISA and the UTEP plaque reduction neutralization test (NY—PRNT) and by the multiple Microsphere Assay (NY-MIA). (DOC)
