# Supplementary material for: Serosurvey for dengue virus infection among pregnant women in the West Nile virus enzootic community of El Paso Texas
Source: PLoS One. 2020 Nov 30;15(11):e0242889. doi: 10.1371/journal.pone.0242889 (PMC7703982; doi:10.1371/journal.pone.0242889)
Supplement: S1 Table — (DOCX) [file pone.0242889.s001.docx]

**S1 Table. Summary of West Nile and dengue virus antibody detected by enzyme-linked immunoassay (ELISA), New York multiplex microsphere assay (NY - MIA) and the University of Texas at El Paso plaque reduction neutralization test (UTEP - PRNT) in 12 plasma samples obtained from 562 mothers at the time of delivery of newborns in the Providence Hospital, El Paso, Texas.**

|  |  | | **Median Fluorescence Intensity Values of samples reactive with West Nile virus envelope and/or West Nile and dengue virus nonstructural antigens** | | | | | | |  | **UTEP PRNT_80_ Titers** | |  |
| --- | --- | --- | --- | --- | --- | --- | --- | --- | --- | --- | --- | --- | --- |
|  | **ELISA IgG Antibodies** | | **WNV-E** | **WNV-NS1** | **WNV-NS5** | **Den 1 NS1** | **Den 2 NS1** | **Den 3 NS1** | **Den 4 NS1** | **NY - MIA** | **Antibody Titers** | | **PRNT_80_** |
| **Sample Code** | **DENV** | **WNV** | **232*** | **212** | **6680** | **808** | **746** | **615** | **405** | **Diagnosis antibody** | **DENV** | **WNV** | **Diagnosis antibody** |
| PMH0004 | 400 | 3200 | 1399 | 596 | 2193 | 251 | 171 | 107.5 | 367 | WNV | <20 | 80 | WNV |
| PMH0012 | 1600 | 6400 | 6484 | 5240 | 1984 | 119 | 197.5 | 936 | 881 | WNV | <20 | 160 | WNV |
| PMH0037 | 400 | 1600 | 1004 | 171 | 459 | 14 | 10 | 41.5 | 17 | Flavivirus Envelope | <20 | 320 | WNV |
| PMH0118 | 6400 | 6400 | 7965 | 8392 | 2429 | 1529 | 1116 | 4684 | 10051 | WNV/DENV | DENV 2 (320) | 320 | WNV/DENV-2 |
| PMH0149 | 1600 | 6400 | 5942 | 2020 | 2963 | 38 | 83 | 1243 | 217 | WNV / DENV | DENV 2 (640) | 640 | WNV/DENV-2 |
| PMH0167 | 1600 | 6400 | 5523 | 1048 | 1300 | 2498 | 2630 | 8207 | 2944 | WNV/DENV | <20 | 1280 | WNV |
| PMH0171 | 1600 | 6400 | 2999 | 913 | 1124 | 23 | 28 | 86 | 170 | WNV | <20 | 1280 | WNV |
| PMH0249 | 1600 | 6400 | 3085 | 1037 | 1113 | 208 | 334 | 253 | 200 | WNV | DENV2 (160) | 1280 | WNV |
| PMH0278 | 1600 | 6400 | 3122 | 462 | 1082 | 26 | 20 | 136 | 71 | WNV | <20 | 1280 | WNV |
| PMH0279 | 400 | 6400 | 2733 | 1484 | 2825 | 32 | 52 | 169 | 140 | WNV | <20 | 1280 | WNV |
| PMH0288 | 1600 | 6400 | 2549 | 863 | 608 | 19 | 23 | 349 | 165 | WNV | DENV 1 (80) | 320 | WNV |
| PMH0411 | 400 | 1600 | 1026 | 367 | 1643 | 205 | 110 | 69 | 61.5 | WNV | <20 | 320 | WNV |

*- Median fluorescence intensity cut-off values; sample intensity values above cutoff value were recorded as antibody positive and below were antibody negative; values positive for WNV envelope (E) protein only were recorded as antibody to flavivirus envelope, values positive for WNV-E and to one or more WNV non-structural proteins were recorded as positive for WNV antibody, and values positive for WNV-E and to one or more DENV non-structural proteins were recorded as positive for DENV antibody, and samples that were reactive for WNV envelope and for one or more WNV non-structural proteins and one or more DENV non-structural proteins were considered positive for antibody to both WNV/DENV.

ELISA cut-off values for DENV IgG antibody = 0.24-0.29, WNV IgG antibody cut off = 0.11 - 0.16, samples positive for both DENV and WNV antibodies with the same antibody titers or less than 4-fold difference = antibody positive to both viruses, virus with 4-fold or greater antibody titer considered antibody positive for the virus with the highest antibody titer.
