# Supplementary material for: Serosurvey for dengue virus infection among pregnant women in the West Nile virus enzootic community of El Paso Texas
Source: PLoS One. 2020 Nov 30;15(11):e0242889. doi: 10.1371/journal.pone.0242889 (PMC7703982; doi:10.1371/journal.pone.0242889)
Supplement: S2 Table — (DOCX) [file pone.0242889.s002.docx]

**S2 Table. Summary of West Nile and dengue virus antibody detected by enzyme-linked immunoassay (ELISA), New York multiplex microsphere assay (NY - MIA) and the University of Texas at El Paso plaque reduction neutralization test (UTEP - PRNT) in 36 plasma samples obtained from mothers at the time of delivery of newborns in the Sierra East Hospital, El Paso, Texas.**

|  |  | | **Median Fluorescence Intensity Values of samples reactive with West Nile virus envelope and/or West Nile and dengue virus nonstructural antigens** | | | | | | |  | **UTEP PRNT_80_ Titers** | |  |
| --- | --- | --- | --- | --- | --- | --- | --- | --- | --- | --- | --- | --- | --- |
|  | **ELISA IgG Antibodies** | | **WNV-E** | **WNV-NS1** | **WNV-NS5** | **Den 1 NS1** | **Den 2 NS1** | **Den 3 NS1** | **Den 4 NS1** | **NY - MIA** | **Antibody Titers** | | **UTEP PRNT_80_** |
| **Sample**  **Code** | **DEN** | **WN** | **232** | **212** | **6680** | **808** | **746** | **615** | **405** | **Diagnosis**  **antibody** | **DENV** | **WNV** | **Diagnosis antibody** |
| SES0002 | 400 | 1600 | 866 | 338.5 | 923 | 28 | 42 | 77 | 29 | WNV | <20 | 40 | WNV |
| SES0031 | 800 | 6400 | 2409 | 985 | 1659 | 20 | 20 | 208 | 144 | WNV | <20 | 320 | WNV |
| SES0032 | 400 | 3200 | 1082 | 1176 | 1278 | 12 | 12 | 70 | 195 | WNV | <20 | 40 | WNV |
| SES0045 | 800 | 3200 | 2487 | 841 | 1438 | 28 | 20 | 96 | 225 | WNV | <20 | 160 | WNV |
| SES0059 | 200 | 6400 | 1401 | 563 | 640 | 81 | 269 | 71 | 58 | WNV | <20 | 640 | WNV |
| SES0068 | 6400 | 1600 | 3346 | 109 | 305 | 7553 | 3894 | 11714 | 20151 | DENV | DENV1 (160),  DENV2 (640),  DENV3 & 4 (40) | 160 | DENV 2 |
| SES0072 | 800 | 6400 | 3355 | 1303 | 1448 | 27 | 37 | 174 | 93 | WNV | <20 | NT | WNV |
| SES0073A | 3200 | 6400 | 6501 | 2570 | 706 | 43 | 178 | 303 | 391 | WNV | <20 | 40 | WNV |
| SES0111 | 6400 | 6400 | 3483 | 168 | 1139 | 4563 | 1447 | 5767 | 2489 | DENV | DENV1 (1280),  DENV2 & 3 (40) | 160 | DENV 1 |
| SES0146 | 400 | <100 | 737 | 262 | 2862 | 488 | 382 | 860 | 345 | Flavivirus  Envelope | <20 | <20 | NEG |
| SES0206 | 6400 | 6400 | 6289 | 1856 | 2219 | 67 | 60 | 412 | 514 | WNV | <20 | 80 | WNV |
| SES0242 | 6400 | 1600 | 2420 | 81 | 461 | 8446 | 1331 | 6304 | 6354 | DENV | DEN1 (640),  DEN2 & 3 (160) | 80 | DENV1 |
| SES0248 | 6400 | 800 | 2675 | 82 | 1355 | 2332 | 1775 | 5808 | 1501 | DENV | DEN1 (160),  DEN2 (640),  DEN3 (160),) | 40 | DENV2 |
| SES0251 | 400 | 3200 | 3325 | 421 | 581 | 29 | 37 | 179 | 38 | WNV | <20 | 160 | WNV |
| SES0297 | 400 | 1600 | NT | NT | NT | NT | NT | NT | NT | WNV | <20 | 640 | WNV |
| SES0412 | 1600 | 6400 | 3687 | 995 | 563 | 12 | 22 | 112 | 36 | WNV | <20 | 160 | WNV |
| SES0426 | 6400 | 6400 | 3194 | 1390 | 2808 | 41 | 42 | 185 | 148 | WNV | DEN2 (160) | 1280 | WNV |
| SES0447 | 6400 | 6400 | 4051 | 62 | 781 | 6981 | 488 | 1829 | 10645 | DENV | DEN1 (160),  DEN2 (320 | <20 | DENV1 & 2 |
| SES0450 | 400 | 6400 | 364 | 81 | 629 | 19 | 29 | 20 | 19 | Flavivirus Envelope | <20 | 1280 | WNV |

*- Median fluorescence intensity cut-off values for test samples equal to or higher representing antigen – antibody reactivity, ELISA cut-off values DENV IgG antibody cut off = 0.24-0.29, WNV IgG antibody cut off= 0.11-0.16, samples positive for both DENV and WNV antibodies with the same antibody titers or less than 4-fold difference = antibody positive to both viruses, virus with 4-fold or greater antibody titer considered antibody positive for the virus with the highest antibody titer.

**S2 Table (continued). Summary of West Nile and dengue virus antibody detected by enzyme-linked immunoassay (ELISA), New York multiplex microsphere assay (NY - MIA) and the University of Texas at El Paso plaque reduction neutralization test (UTEP - PRNT) in 36 plasma samples obtained from 752 mothers at the time of delivery of newborns in the Sierra East Hospital, El Paso, Texas.**

|  |  | | **Median Fluorescence Intensity Values of samples reactive with West Nile virus envelope and/or with West Nile and dengue virus (DEN) nonstructural antigens** | | | | | | |  | **UTEP PRNT_80_ Titers** | |  |
| --- | --- | --- | --- | --- | --- | --- | --- | --- | --- | --- | --- | --- | --- |
|  | **ELISA IgG Antibodies** | | **WNV-E** | **WNV-NS1** | **WNV-NS5** | **Den 1 NS1** | **Den 2 NS1** | **Den 3 NS1** | **Den 4 NS1** | **NY - MIA** | **Antibody**  **Titers** | | **UTEP PRNT_80_** |
| **Sample Code** | **DEN** | **WN** | **232** | **212** | **6680** | **808** | **746** | **615** | **405** | **Diagnosis antibody** | **DENV** | **WNV** | **Diagnosis antibody** |
| SES0475 | 6400 | 6400 | 3081 | 756 | 576 | 22 | 139 | 73 | 51 | WNV | DENV2 (160) | 1280 | WNV |
| SES0477 | 1600 | 6400 | 1791 | 1691 | 889 | 12 | 12 | 50 | 260 | WNV | <20 | 160 | WNV |
| SES0496 | 6400 | 6400 | 4778 | 2192 | 1190 | 29 | 31 | 391 | 179 | WNV | <20 | 640 | WNV |
| SES0500 | 6400 | 400 | 522 | 54 | 636 | 406 | 839 | 2809 | 124 | DENV | DENV2 (320) | <20 | DENV2 |
| SES0625 | 6400 | 6400 | 1703 | 313 | 412 | 31 | 56 | 115 | 31 | WNV | DENV4 (40) | 320 | WNV |
| SES0629 | 6400 | 6400 | 2616 | 1284 | 1257 | 59 | 54 | 175 | 296 | WNV | DENV4 (160) | 1280 | WNV |
| SES0632 | 400 | 6400 | 620 | 179 | 504 | 19 | 15 | 62 | 31 | Flavivirus Envelope | <20 | 1280 | WNV |
| SES0635 | 1600 | 6400 | 2256 | 859 | 2996 | 15 | 122 | 48 | 97 | WNV | <20 | 320 | WNV |
| SES0647 | 1600 | 6400 | 3249 | 1532 | 3716 | 29 | 33 | 123 | 188 | WNV | DENV4 (80} | 320 | WNV |
| SES0654 | 1600 | 6400 | 2343 | 249 | 1053 | 46 | 53 | 55 | 46 | WNV | DENV3 (160), DENV 4 (640) | 320 | WNV/DENV |
| SES0658 | 1600 | 6400 | 1996 | 386 | 1424 | 30 | 24 | 75 | 43 | WNV | DENV3 (160) | 1280 | WNV |
| SES0664 | 6400 | 6400 | 2794 | 1072 | 583 | 36 | 31 | 106 | 76 | WNV | <20 | 1280 | WNV |
| SES0669 | 400 | 6400 | 2343 | 709 | 1027 | 22 | 25 | 200 | 76 | WNV | DENV4 (40) | 160 | WNV |
| SES0670 | 400 | 6400 | 1314 | 490 | 2042 | 14 | 15 | 88 | 85 | WNV | DENV4 (40) | 640 | WNV |
| SES0675 | 400 | 6400 | 4496 | 953 | 1544 | 27 | 28 | 176 | 85 | WNV | <20 | 160 | WNV |
| SES0827 | 1600 | 1600 | 273 | 53 | 658 | 273 | 113 | 461 | 78 | Flavivirus  Envelope | <20 | 320 | WNV |
| SES0836 | 6400 | 6400 | 3649 | 2515 | 1510 | 125 | 85 | 177 | 680 | WNV | <20 | 320 | WNV |

*- Median fluorescence intensity cut-off values for test samples equal to or higher representing antigen – antibody reactivity, ELISA cut-off values DENV IgG cut off = 0.24-0.29, WNV IgG cut off=0.11-0.16, samples positive for both DENV and WNV antibodies with the same antibody titers or less than 4-fold difference = antibody positive to both viruses, virus with 4-fold or greater antibody titer considered antibody positive for the virus with the highest antibody titer.
