# Supplementary material for: Serosurvey for dengue virus infection among pregnant women in the West Nile virus enzootic community of El Paso Texas
Source: PLoS One. 2020 Nov 30;15(11):e0242889. doi: 10.1371/journal.pone.0242889 (PMC7703982; doi:10.1371/journal.pone.0242889)
Supplement: S3 Table — (DOCX) [file pone.0242889.s003.docx]

**S3 Table. Summary of West Nile and dengue virus antibody detected by enzyme-linked immunoassay (ELISA) , New York multiplex microsphere assay (NY - MIA) and the University of Texas at El Paso plaque reduction neutralization test (UTEP - PRNT) in 11 plasma samples obtained from 158 mothers at the time of delivery of newborns in the Sierra Main Hospital, El Paso, Texas.**

|  |  | | **Median Fluorescence Intensity Values of samples reactive with West Nile virus envelope and/or with West Nile and dengue virus nonstructural antigens** | | | | | | |  | **UTEP PRNT_80_ Titers** | |  |
| --- | --- | --- | --- | --- | --- | --- | --- | --- | --- | --- | --- | --- | --- |
|  | **ELISA IgG Antibodies** | | **WNV-E** | **WNV-NS1** | **WNV-NS5** | **Den 1 NS1** | **Den 2 NS1** | **Den 3 NS1** | **Den 4 NS1** | **NY - MIA** | **Antibody Titers** | | **UTEP PRNT_80_** |
| **Sample Code** | **DEN** | **WN** | **232** | **212** | **6680** | **808** | **746** | **615** | **405** | **Diagnosis**  **antibody** | **DENV** | **WNV** | **Diagnosis**  **antibody** |
| SMC0006 | 400 | 6400 | NT | NT | NT | NT | NT | NT | NT | WNV | <20 | 160 | WNV |
| SMC0008 | 1600 | 6400 | 4019 | 1105 | 1194 | 17 | 215 | 74 | 93 | WNV | <20 | 320 | WNV |
| SMC0013 | 800 | 6400 | 964 | 335 | 235 | 19 | 20 | 44 | 26 | WNV | <20 | 320 | WNV |
| SMC0040 | 1600 | 6400 | 2914 | 1378 | 2264 | 39 | 44 | 254 | 333 | WNV | <20) | 80 | WNV |
| SMC0053 | 1600 | 6400 | 1104 | 589 | 944 | 30 | 33 | 62 | 39 | WNV | <20 | 1280 | WNV |
| SMC0055 | 1600 | 1600 | 407 | 50 | 439 | 12 | 10 | 107 | 15 | Flavivirus Envelope | <20 | <20 | Negative (NEG) |
| SMC0067 | 6400 | 1600 | 2517 | 70 | 773 | 910 | 1149 | 4124 | 2195 | DENV | DENV2 (2560) | 1280 | DENV2 |
| SMC0068 | 400 | 1600 | 2894 | 754 | 2055 | 203 | 177 | 147 | 91 | WNV | <20 | 1280 | WNV |
| SMC0070 | 400 | 1600 | 2343 | 593 | 885 | 46 | 238 | 229 | 377 | WNV | <20 | 1280 | WNV |
| SMC0114 | 6400 | 6400 | 5049 | 76 | 1221 | 11128 | 2276 | 8388 | 4198 | DENV | DENV1 (2560), DENV2 (320) | 320 | DENV1 |
| SMC0119 | 200 | <100 | 851 | 33 | 331 | 569 | 149 | 310 | 71 | Flavivirus  Envelope | <20 | <20 | Neg |

*- Median fluorescence intensity cut-off values for test samples equal to or higher representing antigen – antibody reactivity, ELISA cut-off values DENV IgG cut off = 0.24-0.29, WNV IgG cut off=0.11-0.16, samples positive for both DENV and WNV antibodies with the same antibody titers or less than 4-fold difference = antibody positive to both viruses, virus with 4-fold or greater antibody titer considered antibody positive for the virus with the highest antibody titer.
