# Supplementary material for: Serosurvey for dengue virus infection among pregnant women in the West Nile virus enzootic community of El Paso Texas
Source: PLoS One. 2020 Nov 30;15(11):e0242889. doi: 10.1371/journal.pone.0242889 (PMC7703982; doi:10.1371/journal.pone.0242889)
Supplement: S4 Table — (DOC) [file pone.0242889.s004.doc]

**S4 Table. Summary of 28 plasma samples, including 3 from mothers at the Providence Memorial, 18 from mothers at the Sierra East, and 7 from mothers a the Sierra Main hospitals, El Paso, Texas that were negative for antibody by ELISA and the UTEP plaque reduction neutralization test (NY - PRNT) and by the multiple microsphere assay (NY-MIA).**

|  |  | | **Median Fluorscence Intensity Values of samples reactive with nonstructural antigens** | | | | | | |  | **UTEP PRNT_80_ Titers** | |  |  |
| --- | --- | --- | --- | --- | --- | --- | --- | --- | --- | --- | --- | --- | --- | --- |
|  | **ELISA IgG Antibodies** | | **WNV-E** | **WNV-NS1** | **WNV-NS5** | **Den 1 NS1** | **Den 2 NS1** | **Den 3 NS1** | **Den 4 NS1** | **MIA** | **Antibody Titers** | | **UTEP PRNT** |  |
| Sample Code | DEN | WN | 232 | 212 | 6680 | 808 | 746 | 615 | 405 | **Diagnosis** | **DENV** | **WNV** | **Diagnosis** |  |
| PMH008 | <100 | <100 | 211 | 207 | 1086 | 73 | 41 | 348 | 82 | NEG | **<20** | **<20** | NEG |  |
| PMH014 | <100 | <100 | 31 | 79 | 540 | 17 | 11 | 79 | 23 | NEG | **<20** | **<20** | NEG |  |
| PMH0105 | <100 | <100 | 18 | 51 | 425 | 11 | 12 | 61 | 17 | NEG | **<20** | **<20** | NEG |  |
| SES0228 | <100 | <100 | 14 | 78 | 736 | 32 | 14 | 46 | 20 | NEG | <20 | <20 | NEG | |
| SES0232 | <100 | <100 | 71 | 46 | 571 | 38 | 52 | 72 | 16 | NEG | <20 | <20 | NEG | |
| SES0247 | <100 | <100 | 16 | 49 | 399 | 28 | 16 | 47 | 16 | NEG | <20 | <20 | NEG | |
| SES0252 | <100 | <100 | 12 | 53 | 570 | 25 | 30 | 38 | 49 | NEG | <20 | <20 | NEG | |
| SES0255 | <100 | <100 | 23 | 68 | 502 | 29 | 17 | 32 | 17 | NEG | <20 | <20 | NEG | |
| SES0264 | <100 | <100 | 10 | 66 | 1193 | 24 | 44 | 60 | 103 | NEG | <20 | <20 | NEG | |
| SES0273 | <100 | <100 | 8 | 57 | 435 | 21 | 23 | 34 | 20 | NEG | <20 | <20 | NEG | |
| SES0275 | <100 | <100 | 22 | 40 | 617 | 34 | 45 | 54 | 24 | NEG | <20 | <20 | NEG | |
| SES0279 | <100 | <100 | 76 | 71 | 561 | 39 | 24 | 180 | 51 | NEG | <20 | <20 | NEG | |
| SES0282 | <100 | <100 | 17 | 56 | 1148 | 21 | 14 | 49 | 26 | NEG | <20 | <20 | NEG | |
| SES0284 | <100 | <100 | 8 | 32 | 292 | 6 | 6 | 48 | 12 | NEG | <20 | <20 | NEG | |
| SES0299 | <100 | <100 | 18 | 55 | 1118 | 1455 | 615 | 82 | 38 | NEG | <20 | <20 | NEG | |
| SES0402 | <100 | <100 | 13 | 45 | 482 | 16 | 20 | 38 | 19 | NEG | <20 | <20 | NEG | |
| SES0405 | <1000 | <100 | 9 | 51 | 409 | 28 | 19 | 64 | 16 | NEG | <20 | <20 | NEG | |
| SES0407 | <100 | <100 | 12 | 52 | 757 | 19 | 16 | 43 | 14 | NEG | <20 | <20 | NEG | |
| SES0409 | <100 | <100 | 41 | 73 | 334 | 31 | 48 | 56 | 25 | NEG | <20 | <20 | NEG | |
| SES0067 | <100 | <100 | 19 | 53 | 272 | 39 | 30 | 49 | 28 | NEG | <20 | <20 | NEG | |
| SES0249 | <100 | <100 | 25 | 32 | 408 | 44 | 56 | 66 | 52 | NEG | <20 | <20 | NEG | |
| SMC0041 | <100 | <100 | 37 | 40 | 383 | 28 | 36 | 27 | 16 | NEG | <20 | <20 | NEG | |
| SMC0059 | <100 | <100 | 7 | 35 | 913 | 33 | 7 | 52 | 37 | NEG | <20 | <20 | NEG | |
| SMC0063 | <100 | <100 | 15 | 66 | 894 | 28 | 17 | 96 | 28 | NEG | <20 | <20 | NEG | |
| SMC0069 | <100 | <100 | 10 | 53 | 475 | 26 | 25 | 80 | 24 | NEG | <20 | <20 | NEG | |
| SMC0073 | <100 | <100 | 29 | 54 | 1224 | 28 | 25 | 62 | 24 | NEG | <20 | <20 | NEG | |
| SMC0076 | <100 | <100 | 8 | 59 | 1377 | 9 | 8 | 345 | 18 | NEG | <20 | <20 | NEG | |
| SMC0451 | <100 | <100 | 43 | 76 | 587 | 150 | 168 | 149 | 211 | NEG | <20 | <20 | NEG | |
